# Tumour habitat-based radiomics analysis enhances the ability to predict prostate cancer aggressiveness with biparametric MRI-derived features

**DOI:** 10.3389/fonc.2025.1504132

**Published:** 2025-03-17

**Authors:** Mengjuan Li, Ning Ding, Shengnan Yin, Yan Lu, Yiding Ji, Long Jin

**Affiliations:** Department of Radiology, Suzhou Ninth Hospital Affiliated to Soochow University, Suzhou, Jiangsu, China

**Keywords:** habitat, prostate cancer, classification, aggressiveness, machine learning

## Abstract

**Objective:**

The purpose of this study was to develop three predictive models utilising clinical factors, radiomics features, and habitat features, to distinguish between nonclinically significant prostate cancer (csPCa) and clinically significant PCa (non-csPCa) on the basis of biparametric MRI (bp-MRI).

**Methods:**

A total of 175 patients were enrolled, including 134 individuals with csPCa and 41 with non-csPCa. The clinical model was developed using optimal predictive factors obtained from univariable logistic regression and modelled through a random forest approach. Image acquisition and segmentation were performed first in the creation of both the radiomics model and the habitat model. The K-means clustering algorithm was then used exclusively for habitat generation in the development of the habitat model. Finally, feature selection and model construction were performed for both models. Model comparison and diagnostic efficacy assessment were conducted through receiver operating characteristic curve analysis, decision curve analysis (DCA), and calibration curve analysis.

**Results:**

The habitat model outperformed both the radiomics model and the clinical model in distinguishing csPCa from non-csPCa patients. The AUC values of the habitat model in the training and test sets were 0.99 and 0.93, respectively. Furthermore, DCA and the calibration curves highlighted the superior clinical utility and greater predictive accuracy of the habitat model in comparison with the other two models.

**Conclusion:**

We developed a habitat-based radiomics model with a greater ability to distinguish between csPCa and non-csPCa on the basis of bp-MRI than a traditional radiomics model and clinical model. This introduces a novel approach for assessing the heterogeneity of PCa and offers urologists a quantitative, noninvasive method for preoperatively evaluating the aggressiveness of PCa.

## Introduction

Prostate cancer (PCa) is one of the most common types of cancer among men worldwide. The incidence has been increasing annually since 2015, and as of 2024, PCa accounted for nearly one-third of all cancers, with nearly three times the prevalence of the second most common cancer, lung and bronchial cancer. It is also the second leading cause of cancer death among men; a cancer statistics report for 2024 revealed that out of 322,800 patients with PCa, 35,250 (approximately 11%) died (1). The 2020 guidelines emphasise the urgent need to distinguish between nonclinically significant PCa, which typically has a slow progression and is best managed with active surveillance, and the more aggressive, clinically significant PCa, which demands immediate intervention owing to its rapid progression (2). Timely identification of PCa and precise evaluation of its aggressiveness are paramount for tailoring the most effective treatment strategy to increase survival rates and improve patient outcomes, even in the face of the diagnostic challenge of overlapping clinical symptoms with benign prostate conditions.

Traditional PCa diagnostic methods, such as the serum prostate-specific antigen (PSA) index and transrectal ultrasound-guided needle biopsy (TRUS), have significant drawbacks. TRUS, while the standard for assessing cancer invasiveness, is itself invasive, can cause discomfort, and may not accurately reflect the severity of cancer. Overreliance on the PSA level, meanwhile, can lead to overdiagnosis and unnecessary procedures, whereas biopsies pose risks of pain and infection (3, 4). Additionally, some patients with benign prostatic hyperplasia (BPH) may undergo unnecessary biopsies, and there is a chance of a false negative result for certain PCa patients. In light of these issues, more accurate and less invasive diagnostic methods are urgently needed. MRI, specifically multiparametric MRI (mp-MRI), has become an essential tool for detecting, locating, and grading PCa (5, 6). The introduction of streamlined biparametric MRI (bp-MRI), which includes T2-weighted imaging (T2WI) and diffusion-weighted imaging (DWI)—particularly apparent diffusion coefficient (ADC) mapping—as per the PI-RADS v2.1, is a step towards a more efficient diagnostic approach (7). Recent studies have demonstrated that, compared to traditional imaging methods, bp-MRI offers superior diagnostic accuracy for PCa. It also simplifies the imaging process, thereby saving time and resources, reducing patient discomfort, and avoiding overdiagnosis (7, 8). This innovation represents a significant shift from traditional methods, marking a new chapter in the early and precise diagnosis of PCa.

Traditional MRI interpretation often relies on radiologists’ subjective interpretations, leading to inconsistencies due to varying levels of expertise and a lack of objectivity. However, the integration of machine learning (ML) has transformed the field by introducing a more systematic and quantitative method for analysing medical images. Radiomics, an emerging imaging field, excels in this role by extracting numerous features from medical images and converting them into comprehensive datasets. These features encompass data about tumour heterogeneity and the microenvironment (9, 10), enabling a more accurate assessment of tumour traits and treatment response (11–13). Unlike previous methods, a novel radiomic-based approach divides whole tumours into subregions known as habitats, which contain voxels with similar attributes and consistent tumour biology (14). This methodology has significantly enhanced the quantification of intratumoural heterogeneity (15, 16).

To our knowledge, few studies have aimed to conduct habitat-based radiomics analyses to predict PCa aggressiveness on the basis of bp-MRI. In this research, we employed a new radiomics approach to identify imaging biomarkers within the whole tumour region and subregional zones. We established and validated three predictive models based separately on clinical factors, radiomics features, and habitat features to distinguish between csPCa and non-csPCa.

## Materials and methods

### Patient selection and clinicopathological information

**Figure 1:**
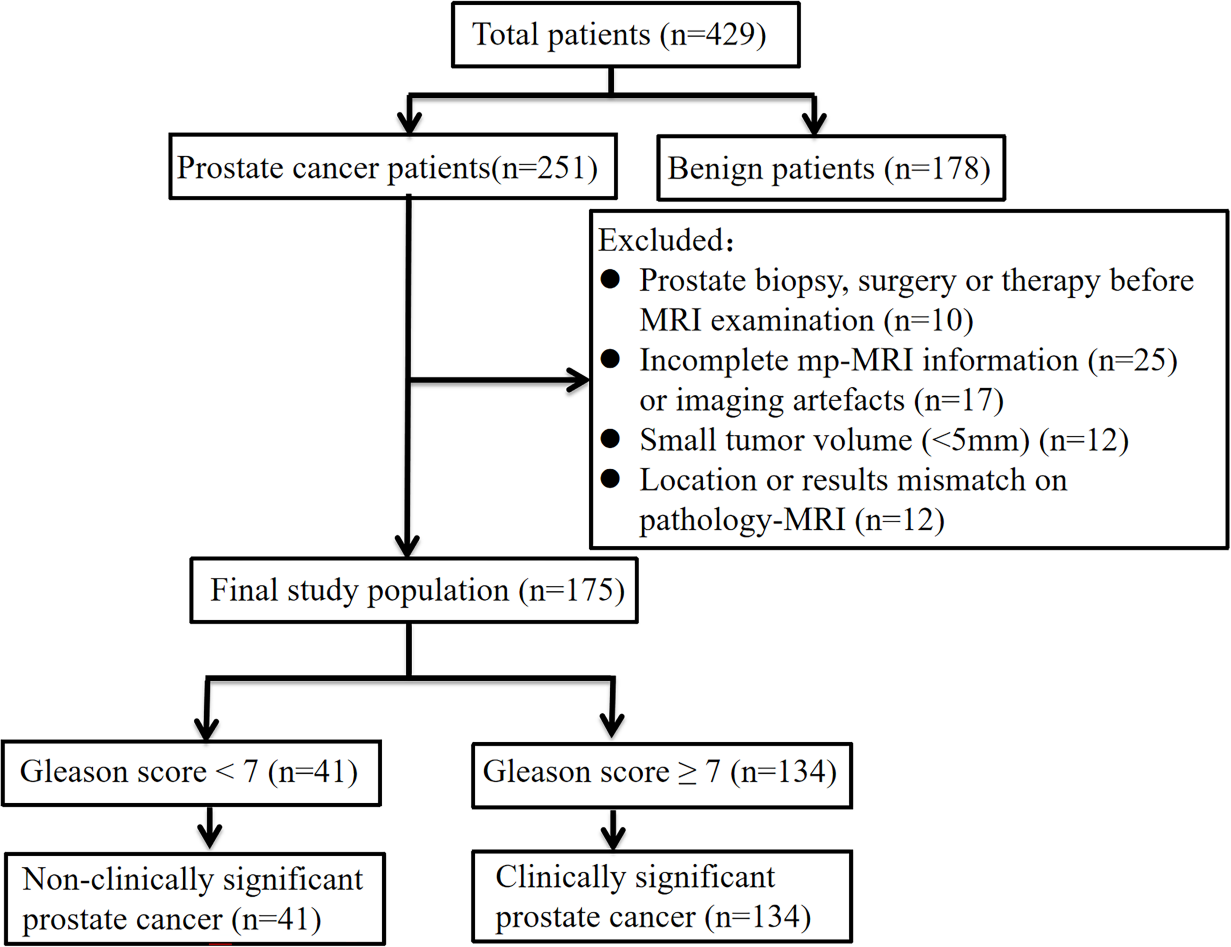
Flowchart of patient selection.

This retrospective study was approved by the Institutional Ethics Committee of Suzhou Ninth Hospital Affiliated to Soochow University (No. KYLW2024-052-01), who waived the requirement for written informed consent. A total of 429 patients in our hospital who underwent 3.0T MRI examination due to elevated PSA or clinical symptoms (such as frequent urination, urgent urination, pain in urine, etc.) from January 2019 to January 2023 and who had pathological results were included in our research. The details of the patient selection and grouping processes are shown in **Figure 1**. Finally, 175 patients, including 134 with csPCa and 41 with non-csPCa, were enrolled and randomly divided into training and test sets at a 7:3 ratio. All patients underwent TRUS-guided systematic prostate biopsy.

The collected clinical factors for the patients included age, prostate volume (PV), total PSA (tPSA), free PSA (fPSA), the ratio of fPSA to tPSA (f/t PSA), and the PSA density (PSAD). The PV was calculated by multiplying the width, length, and height of the prostate on T2WI by 0.52 (17). The PSAD was calculated as tPSA/PV.

### Workflow of radiomics analysis

**Figure 2:**
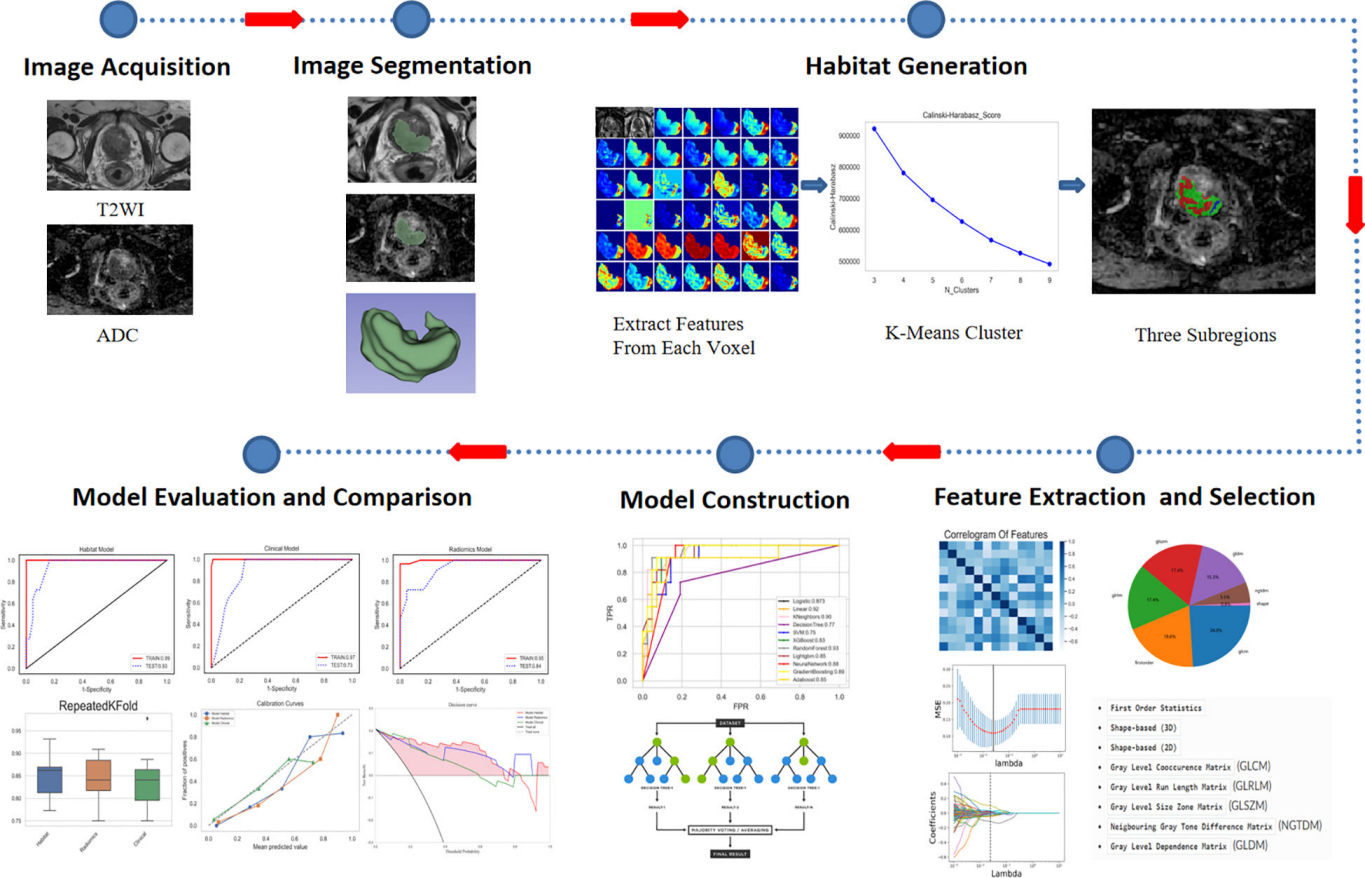
Workflow of the radiomics analysis.

Radiomics analysis was executed through a series of steps, including image acquisition and segmentation, feature extraction, feature selection, and model construction. The entire flowchart for the workflow employed in this research is presented in **Figure 2**.

### Image acquisition and segmentation

All patients underwent scans on a 3.0-T MRI device (GE Discovery MR750, USA). The scan sequences included sagittal and axial T2WI, T1-weighted imaging (T1WI), fat-suppressed T2WI, DWI (b values of 50 and 1,400 sec/mm ^2^) and dynamic contrast-enhanced MRI (DCE-MRI). At the end of the scan, the machine automatically generated an ADC map on the basis of the DWI sequence. In this study, a bp-MRI sequence that includes axial T2WI and ADC mapping was selected as recommended by the PI-RADS v2.1 due to its combination of accuracy and convenience. The parameters of the T2WI sequence were as follows: repetition time (TR) = 3000 ms, echo time (TE) = 100 ms, thickness = 3 mm, gap = 0 mm, field of view (FOV) = 220*220 mm, matrix = 276*238, and number of excitations (NEX) = 3. The parameters of the DWI sequence were as follows: TR = 6000 ms, TE = 77 ms, thickness = 3 mm, gap = 0 mm, FOV = 260*260 mm, matrix = 104*126, and NEX = 2. To bolster the reliability of the medical image analyses, a series of preprocessing steps were implemented prior to image segmentation. First, the PyRadiomics package (version 3.1.0, https://pyradiomics.readthedocs.io/en/latest/) was employed to perform N4BiasFieldCorrection on the MR images, effectively correcting low-frequency bias fields and eliminating background inhomogeneity to increase contrast and improve accuracy (18). Second, each original image was resampled to a size of 1 * 1 * 1 mm^3^. Third, the ADC image was registered to the T2W image for spatial alignment in terms of anatomical structure for subsequent analysis and diagnosis. The registration process involved the use of the “SlicerElastix” plugin, an extension module integrated into the 3D Slicer software. This plugin leverages the Elastix medical image registration toolkit, providing robust image registration capabilities. Through the SlicerElastix plugin, medical experts can achieve high-precision image registration, which is crucial for the diagnosis and treatment planning of pathologies such as prostate cancer. This structure-based spatial alignment ensures that image data acquired under different imaging conditions can be accurately compared and evaluated. A seasoned radiologist with a 10 years of expertise subsequently meticulously delineated the region of interest (ROI) corresponding to the lesion on T2WI layer by layer with 3D-Slicer software (version 5.6.2, https://www.slicer.org/); all ROIs were then consolidated into a volume of interest (VOI). In instances of doubt or uncertainty, the radiologist consulted with a fellow senior radiologist with 12 years of experience to ensure that the ROIs were precisely delineated. Importantly, both physicians were blinded to the patients’ clinical and pathological data throughout the evaluation, thereby increasing the objectivity and impartiality of the study.

### Habitat generation

Initially, we extracted 40 features from each voxel within the lesion area, including first-order statistics and grey-level co-occurrence matrix (GLCM) features. The first-order features, derived directly from the distribution of image pixel intensities, include statistical measures such as the mean, variance, and standard deviation, which provide a description of the central tendency, dispersion, and heterogeneity, respectively, of the distribution of the image brightness. The GLCM, on the other hand, captures textural features of the image by analysing the relationships between pixel intensities and their immediate neighbours, thereby revealing microstructural textural characteristics such as coarseness, directionality, and uniformity. These features were then integrated to form a comprehensive feature vector, each of which represents a unique set of properties for the corresponding voxel.

We subsequently conducted an in-depth analysis of these feature vectors with the K-means clustering algorithm, a classical unsupervised learning technique (19). Through an iterative process, the K-means algorithm partitions the feature vectors into K clusters, each defined by its centroid—the mean of all points within the cluster. The algorithm aims to minimise the distance between voxels within a cluster and the cluster centre, thereby allowing identification of patterns and structures within the data. Determining the optimal number of clusters during the clustering process is crucial; to aid in this determination, we employed the Calinski–Harabasz (CH) index, which evaluates the ratio of intracluster compactness to intercluster separation, assisting in identifying the most discriminative number of clusters (20). This approach enables the determination of the best subregion division.

### Feature extraction and selection

The PyRadiomics tool was used to extract features including first-order, shape (including 2D and 3D), GLCM, grey level run length matrix (GLRLM), grey level size zone matrix (GLSZM), grey level dependence matrix (GLDM), and neighbouring grey tone difference matrix (NGTDM) features, all in accordance with the guidelines of the Imaging Biomarker Standardization Initiative (IBSI). While many features were identified, not all of them were valuable in distinguishing csPCa from non-csPCa. Therefore, three feature dimension reduction methods were employed to determine the most discriminative set of features. First, we used mutual information-based feature selection, which assesses the relevance between features and the classification target as well as the redundancy among features to select the most beneficial subset of features for classification. Through three steps—feature subset generation, subset evaluation, and stopping criteria—it effectively reduces the dimensionality of the features, thereby increasing the efficiency and accuracy of the resulting classification model. Second, the maximum relevance minimum redundancy (mRMR) method was used to eliminate irrelevant and redundant features. Third, we utilised the least absolute shrinkage and selection operator (LASSO) to meticulously select the most impactful features for the models, ensuring a streamlined and less sparse structure and mitigating the propensity for overfitting. By carefully fine-tuning the regularisation parameter λ, LASSO effectively makes the regression coefficients of extraneous features zero. The optimal λ was identified through 10-fold cross-validation, with the goal of achieving the lowest possible mean square error. Finally, the Pearson correlation coefficient was used to eliminate features with high consistency, that is, those with a correlation coefficient greater than 0.8.

### Model construction and performance evaluation

To establish the clinical model, univariable logistic regression analysis was used to obtain clinical features with P values<0.05, and then the random forest method was used to establish the model. To establish the radiomics and habitat models, numerous machine learning models were applied, including logistic regression, linear regression, k-nearest neighbour (KNN), decision tree, support vector machine (SVM), eXtreme Gradient Boosting (XGBoost), random forest (RF), light gradient boosting machine (LightGBM), neural network, gradient boosting machine (GBM), and AdaBoost.

The diagnostic performance of the clinical, radiomics and habitat models was assessed and compared from multiple perspectives with three distinct approaches: receiver operating characteristic (ROC) curve analysis, decision curve analysis (DCA), and calibration curve analysis. ROC curve analysis included calculation of the area under the curve (AUC), accuracy, sensitivity and specificity were computed. Calibration curves were plotted to assess the accuracy of the models’ predictions. Finally, the clinical applicability of the predictive models was evaluated with DCA (21).

### Statistical analysis

The data were statistically analysed in R language (version 4.3.0, https://www.r-project.org) and Python language software (version 3.9, https://www.python.org). The Shapiro-Wilk test was used to assess the normality of the distributions of the variables. Variables conforming to a normal distribution are described using the mean and standard deviation, whereas those not conforming to a normal distribution are described with the median (Q1, Q3). The Mann-Whitney U test was used to assess differences between two groups. Categorical data are presented as counts and were compared between groups with the chi-square test. Comparisons of the AUCs were conducted with the DeLong test. *P* < 0.05 was considered to indicate statistical significance.

## Results

### Patient characteristics

A total of 175 patients (134 with csPCa and 41 with non-csPCa) were included in this research. All the subjects were then divided into training (140) and test (35) sets via stratified random sampling at a 7:3 ratio. The characteristics of all patients are listed in **Table 1**.

**Table 1:** Patient characteristics.

Characteristics	Total (n=175)	Clinically significant PCa (n = 134)	Nonclinically significant PCa (n = 41)	*P* value
Age, years, median (Q1, Q3)	74 (68, 80)	74 (67, 80.8)	74 (71, 78)	0.982
PV, mL, median (Q1, Q3)	39.8 (28, 56.5)	38.8 (27.4, 58.4)	40.6 (32, 49.3)	0.857
tPSA, ng/mL, median (Q1, Q3)	39.8 (28, 56.5)	55.1 (20.4, 113.1)	12.1 (8.8, 18)	< 0.001
fPSA, ng/mL, median (Q1, Q3)	3.2 (1.3, 10.7)	5.3 (1.9, 16.5)	1.2 (0.9, 2.2)	< 0.001
PSAD, ng/mL/mL, median (Q1, Q3)	1 (0.4, 2.1)	1.4 (0.6, 2.6)	0.3 (0.2, 0.6)	< 0.001
f/tPSA, %, median (Q1, Q3)	1 (0.4, 2.1)	0.1 (0.1, 0.2)	0.1 (0.1, 0.2)	0.748
Gleason Score				
3 + 3 = 6		–	41	–
3 + 4/4 + 3 = 7		56	–	
4 + 4 = 8		41	–	
4 + 5/5 + 4 = 9		28	–	
5 + 5 = 10		9	–	

PCa, prostate cancer; PV, prostate volume; tPSA, total prostate-specific antigen; fPSA, free PSA; f/tPSA, ratio of free-to-total PSA; PSAD, PSA density.

### Clinical model

Univariable logistic analysis revealed that tPSA, fPSA, and PSAD were significant factors for predicting csPCa (P<0.05). The results of the univariable logistic regression analyses are presented in **Supplementary Table S1**. The clinical model was then established with the random forest method on the basis of the selected clinical features.

### Subregion cluster and feature selection

**Figure 3:**
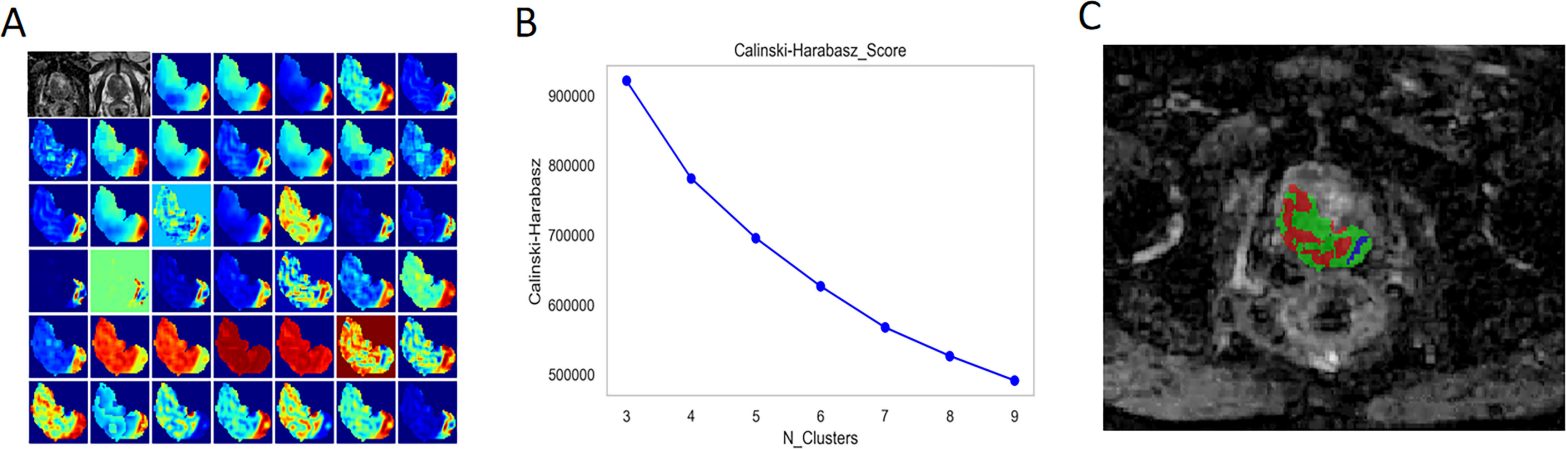
Habitat Generation Process. **(A)** A total of 40 radiomic features were extracted from each voxel within the lesion on T2-weighted imaging (T2WI) and apparent diffusion coefficient (ADC) maps, in addition to the T2WI and ADC values themselves, resulting in a feature vector matrix comprising 42 features for K-means clustering analysis. **(B)** The optimal number of cluster centres was determined to be three based on the Calinski-Harabasz (CH) index, which was subsequently utilised for subregional division of each lesion **(C)**.

Forty radiomic features were extracted from each voxel within the lesion on the T2WI and ADC maps, along with the values of T2WI and ADC, for a total of 42 features that were then used to form a feature vector matrix for K-means clustering analysis. According to the CH value, the optimal number of cluster centres was three, which was then used for the subregional division of each lesion (**Figure 3**).

For the habitat model, a total of 10,004 radiomic features were extracted from the different subregions of the lesions outlined on the T2W and ADC images. After applying the four feature selection methods—mutual information-based feature selection, mRMR, LASSO, and Pearson correlation analysis—13 features were ultimately selected for model construction (**Supplementary Figure S1**). For the radiomics model, a total of 3668 radiomic features were extracted from the different subregions of the lesions outlined on the T2W and ADC images. After the same four feature selection methods were applied, 8 features were ultimately selected for model construction (**Supplementary Figure S2**).

### Model construction and performance comparison

**Figure 4:**
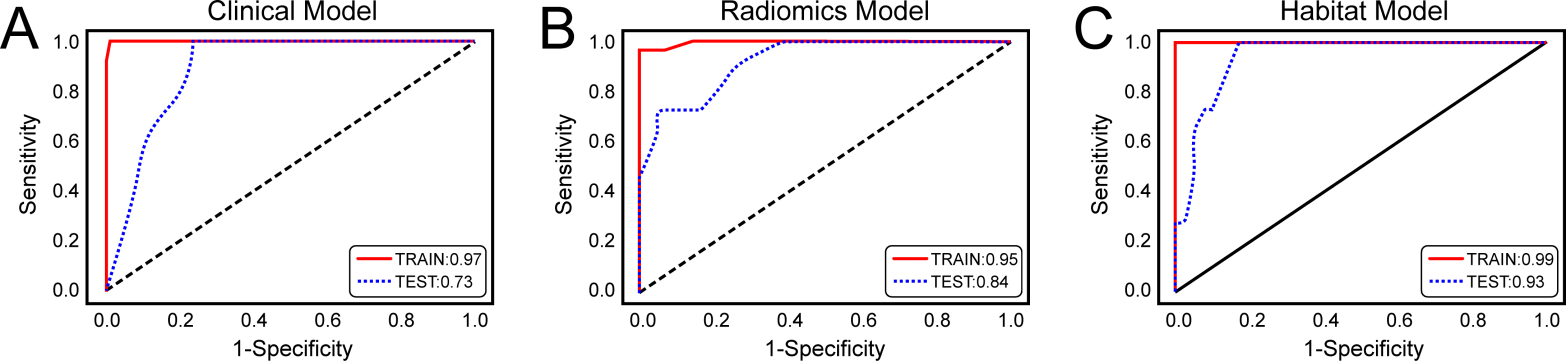
Receiver Operating Characteristic (ROC) Curves of three models for predicting clinically significant prostate cancer. **(A)** ROC curves of the clinical model; **(B)** ROC curves of the radiomics model; **(C)** ROC curves of the habitat model.

**Figure 5:**
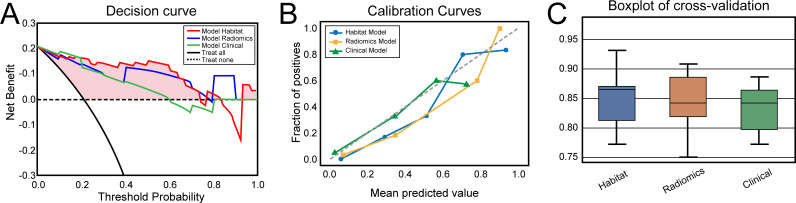
**(A)** Decision curves of the clinical, radiomics and habitat models for predicting clinically significant prostate cancer. **(B)** Calibration curves of the clinical, radiomics and habitat models for predicting clinically significant prostate cancer. **(C)** Boxplots of the five-time four-fold cross-validation results for the clinical, radiomics, and habitat models in predicting clinically significant prostate cancer.

On the basis of the analysis of prediction performance (**Supplementary Figure S3**), the radiomics model and habitat model were constructed with RF. **Figure 4** shows the ROC curves for the clinical model, radiomics model, and habitat model, while **Table 2** provides the details of their ROC-related metrics, including the AUC value, accuracy, sensitivity, specificity, and F1 score. These metrics offer a comprehensive assessment of the diagnostic efficacy of the constructed models. In the test set, the AUC value of the habitat model was 0.93, which was greater than that of the radiomics model and the clinical model. Additionally, DCA indicated that the habitat model offered greater clinical benefits than the other two models did (**Figure 5A**). The calibration curves suggested that the habitat model had better precision than the other models (**Figure 5B**). The cross-validation boxplot also indicates that the habitat model exhibits superior stability and reliability compared to the other models (**Figure 5C**).

**Table 2:** AUC values of the clinical, radiomics and habitat models in the prediction of prostate cancer aggressiveness.

Models		AUC (95% CI)	Accuracy	Sensitivity	Specificity	F1 score
Clinical model	training	0.97 (0.92-1.00)	0.98	0.93	1.00	0.97
	test	0.73 (0.54-0.91)	0.83	0.55	0.90	0.57
Radiomics model	training	0.95 (0.89-1.00)	0. 98	0.90	1.00	0.95
	test	0.84 (0.69-0.99)	0.91	0.73	0.95	0.76
Habitat model	training	0.99 (0.98-1.00)	0.99	1.00	0.99	0.98
	test	0.93 (0.82-1.00)	0.94	0.91	0.95	0.87

AUC, area under the receiver operating characteristic curve; 95% CI, 95% confidence interval; T2WI, T2-weighted imaging; ADC, apparent diffusion coefficient.

## Discussion

In this study, we established and validated three predictive models based separately on clinical factors, radiomics features, and habitat features to assess the aggressiveness of PCa on the basis of bp-MRI. Our results indicated that the habitat model outperformed both the radiomics model and the clinical model in distinguishing csPCa from non-csPCa, with AUC values in the training and test sets of 0.99 and 0.93, respectively. Furthermore, the DCA and calibration curves highlight the superior clinical utility and enhanced accuracy, respectively, of the habitat model with respect to the other two models.

PCa of varying pathological grades exhibit differences at the cellular level. Although rich in information, medical imaging often depends on radiologists’ subjective interpretations and visual acuity, making it challenging to assess pathological grade. Previous studies have indicated that radiomics, a high-throughput analytical technique, can extract textural features from images and convert them into usable data, offering certain value in qualitatively assessing the invasiveness of PCa. Gong et al. developed a 2D model that achieved a C-index of 0.728 and an AUC of 0.794, showing stable key features and robust performance, even in the presence of a 2 mm deviation in segmentation boundaries. The model effectively identified prostate pathology and Gleason scores with radiomic features extracted from 2D prostate-gland MR images (22). Jin et al. developed an integrated clinical-radiomic model that effectively identified csPCa in PI-RADS 3 lesions, achieving an AUC of 0.88, potentially reducing unnecessary biopsies and enhancing the quality of life of the patients (23). Shu et al. reported that a machine learning model constructed using an RF classifier exhibited the best overall predictive performance, with an AUC value of 0.89 for the high-risk group, indicating that machine learning methods based on MR radiomics could be promising tools for accurately stratifying PCa risk (24). The results of this study are similar to those of the aforementioned research; the traditional radiomics model constructed on the basis of bp-MRI achieved AUC values of 0.95 and 0.84 in the training and test sets, respectively, indicating that the model has certain predictive value for the aggressiveness of PCa.

Tumour heterogeneity refers to differences in gene expression, protein levels, and biological behaviours within a tumour and can be divided into spatial heterogeneity and temporal heterogeneity (25). Spatial heterogeneity describes the complexity of the tumour architecture, particularly the interactions between tumour cells, immune cells, and stromal cells, through which the tumour obtains nutrients and growth factors via neovascularisation and regional hypoxia (26). Temporal heterogeneity describes the dynamic process of tumour formation, evolution, and metastasis (27). This heterogeneity has a significant impact on tumour growth, invasiveness, treatment response, and prognosis (28, 29). Traditional radiomics studies often focus on aggregates of imaging features of the entire tumour, which may not fully capture the extent of intratumoural heterogeneity (30). Compared with traditional whole-tumour radiomics, habitat imaging focuses on subregional imaging, providing better quantification of tumour subregions that is better related to tumour growth or invasiveness. By analysing multiple subregions of the tumour and their interrelationships, this method explicitly considers spatial heterogeneity and therefore may provide more precise information for tumour diagnosis and clinical decision-making (31–33).

Several recent studies have indicated that habitat imaging provides valuable insights into the qualitative characteristics of tumours, the prediction of their invasiveness, and the evaluation of treatment efficacy. Prior et al. utilised quantitative MRI and CT imaging techniques to uncover biological correlations within the tumour microenvironment, highlighting the value of habitat imaging in characterising tumour heterogeneity. By employing unsupervised clustering models and certain radiomic features, diverse phenotypes within the tumour, such as cellularity, vascularisation, and necrosis, were explored, revealing a close association between these characteristics and tumour aggressiveness (34). Huang et al. identified habitat-based radiomic features to assess the immediate response of patients with colorectal cancer lung metastases following radiofrequency ablation treatment. They extracted radiomic features from tumour, peritumoural, and specific habitat regions, and the final model combined habitat features and those extracted from a 5-mm peripheral zone, demonstrating the best performance in an independent test set with an AUC of 0.870 (35). Wang et al. utilised PET/CT imaging to extract radiomic features from the entire tumour region and features derived from the habitat technique to predict the Ki-67 status in high-grade serous ovarian cancer. The findings demonstrated that, compared with textural features extracted from the whole tumour region, textural features extracted with the habitat method more effectively predicted the Ki-67 status, demonstrating potential as a biomarker to supersede Ki-67 itself (36). Parra et al. presented a comprehensive methodology for quantifying csPCa detected by radiology, employing DCE-derived habitat analysis and assessing both DCE and ADC features. The resulting model had excellent precision in detecting csPCa, exhibiting robust accuracy across two distinct institutional datasets (37). While these studies were exploratory in nature and require larger sample sizes to validate their findings, they nevertheless demonstrate significant potential in the study of tumour heterogeneity, providing new directions for future cancer research and therapy. The habitat imaging approach used in this study emphasises the importance of subregional cluster analysis in understanding the heterogeneity of PCa. After optimising the number of clusters, we identified three spatially distinct habitats with K-means clustering. As anticipated, following dimensionality reduction, the radiomic features extracted from these unique subregions were used to construct a habitat model that provided valuable information for assessing the aggressiveness of PCa. The habitat model achieved an AUC of 0.93 in the test set, outperforming the radiomics model (AUC = 0.84). This superior performance may be attributed to the subregional analysis employed by the habitat model, which captures the heterogeneity within the tumour and thereby enhances the accuracy of predicting csPCa.

Our study has several limitations. First, this was a single-centre retrospective study with a small sample size and potential selection bias. Therefore, we will collect larger samples and conduct multicentre prospective studies to validate the findings and ensure their generalisability. Second, the machine learning methods utilised in this study relied on manual segmentation, which may introduce subjective bias. In the future, we will focus on automatic segmentation techniques to increase the objectivity of our results. Finally, the uneven distribution of samples in this study may have affected model generalisability, primarily because we observed that the incidence of non-csPCa was significantly lower than that of csPCa during the case collection process. We attempted to mitigate this issue by employing stratified random sampling, but validation of model robustness with a larger and more diverse sample is still necessary. In addition, we will explore the potential of extending habitat-based radiomics to multiparametric MRI to further enhance performance in the future.

## Conclusion

We developed a habitat-based radiomics model that, compared with the traditional radiomic model and clinical model, better distinguished between csPCa and non-csPCa on the basis of bp-MRI. Our study demonstrates a novel approach for assessing the heterogeneity of PCa and offer urologists a quantitative, noninvasive method for preoperatively evaluating the aggressiveness of PCa, thereby reducing unnecessary biopsies and improving patients’ quality of life.

## Data Availability

The original contributions presented in the study are included in the article/**Supplementary Material**. Further inquiries can be directed to the corresponding authors.
