# Supplementary material for: Tumour habitat-based radiomics analysis enhances the ability to predict prostate cancer aggressiveness with biparametric MRI-derived features
Source: Front Oncol. 2025 Mar 17;15:1504132. doi: 10.3389/fonc.2025.1504132 (PMC11955456; doi:10.3389/fonc.2025.1504132)

**Supplementary Method**

***Table S1* Univariate logistic analysis results**

| Clinical factors | OR (95% CI) | *P* value |
| --- | --- | --- |
| Age | 1.01 (0.98, 1.05) | 0.66 |
| PV | 1.00 (0.98, 1.02) | >0.99 |
| tPSA | 0.95 (0.92, 0.97) | <0.001 |
| fPSA | 0.70 (0.56, 0.83) | <0.001 |
| PSAD | 0.12 (0.04, 0.27) | <0.001 |
| f/t PSA | 0.04 (0.00, 1.12) | 0.10 |

OR, odds ratio; CI, Confidence interval; tPSA, total prostate-specific antigen; fPSA, free PSA; PV, prostate volume; PSAD, PSA density; f/tPSA, ratio of free-to-total PSA.

Fig. S1 For the habitat model, a total of 10004 radiomic features were extracted from the different sub-regions of the lesions outlined on T2WI and ADC images. After applying four feature selection methods, mutual information-based feature selection for classification, mRMR, LASSO, and the Pearson correlation coefficient, 13 features were ultimately selected.


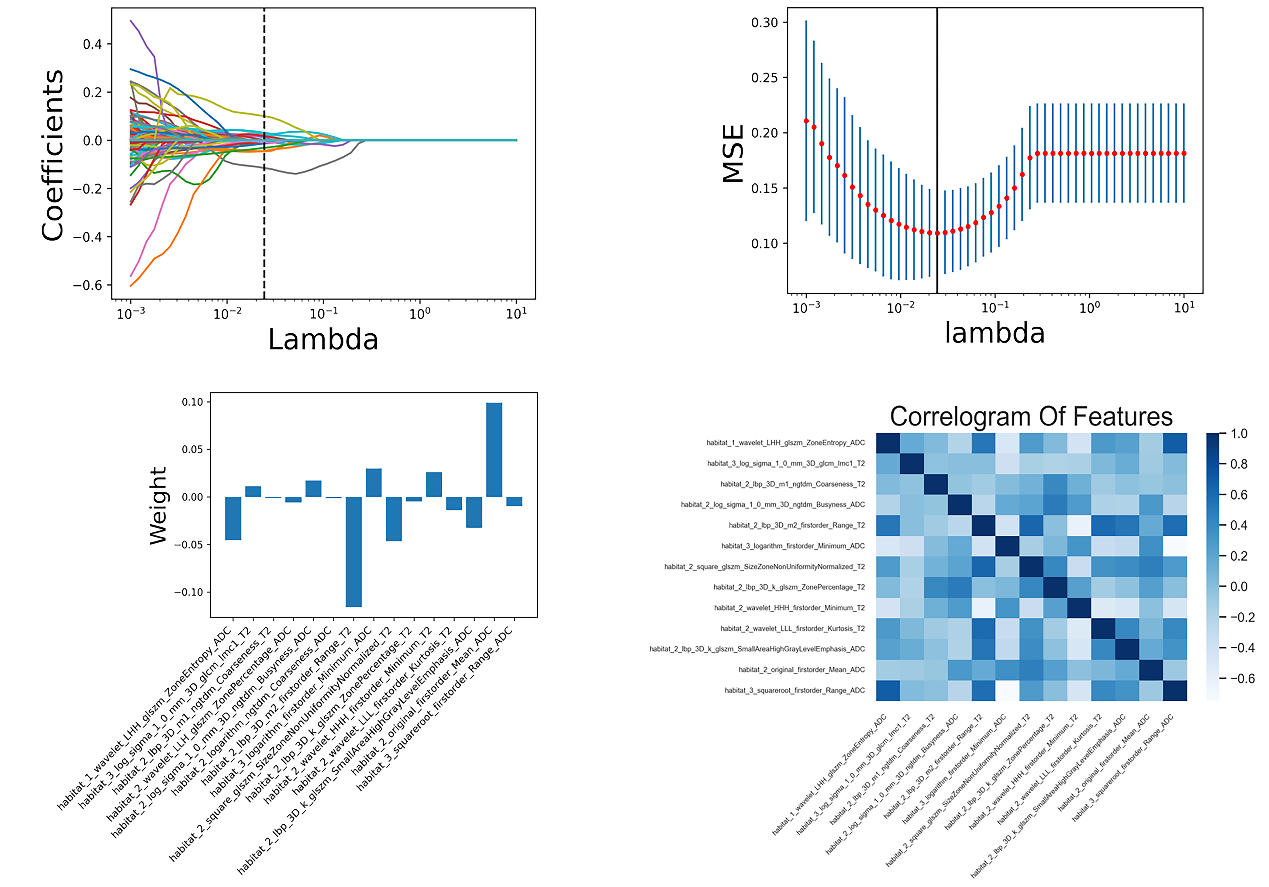


Fig. S2 For the radiomics model, a total of 3668 radiomic features were extracted from the whole lesions outlined on T2WI and ADC images. After applying four feature selection methods, mutual information-based feature selection for classification, mRMR, LASSO, and the Pearson correlation coefficient, 8 features were ultimately selected.


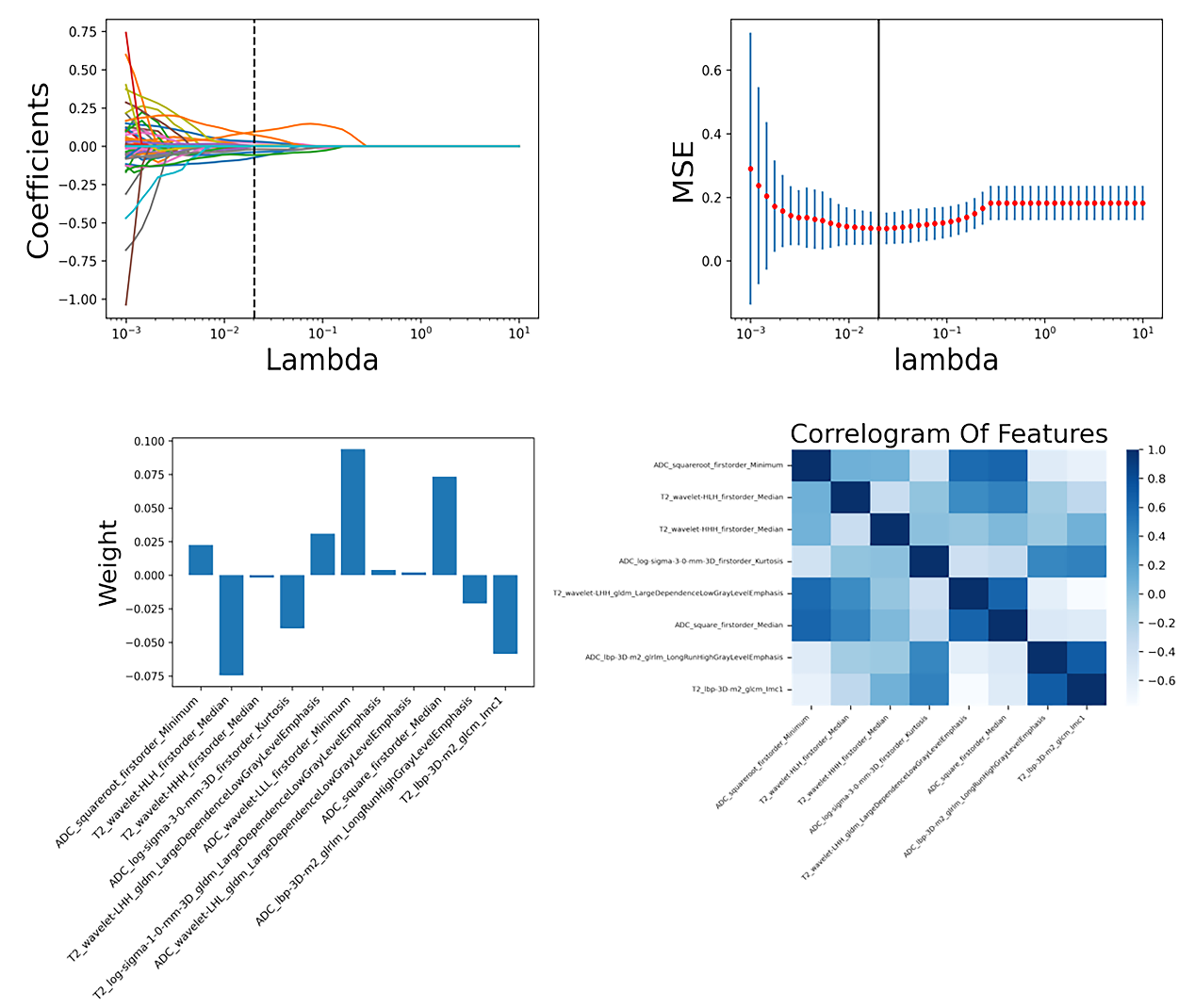


Fig. S3 For the establishment of the radiomics and habitat models, many machine learning models are applied, including: Logistic Regression, Linear Regression, K-nearest Neighbor (KNN), Decision Tree, Support Vector Machine (SVM), eXtreme Gradient Boosting (XGBoost), Random Forest (RF), Light Gradient Boosting Machine (LightGBM), Neural Network, Gradient boosting Machine (GBM), Adaboost.


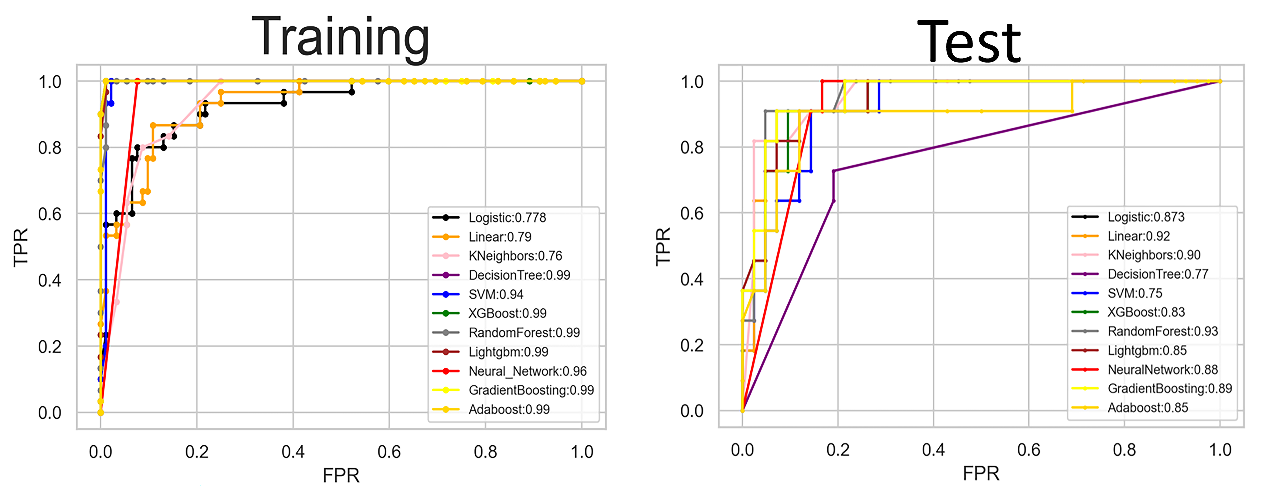

Supplement: Supplementary file 1 [file DataSheet1.docx]
